# Population pharmacokinetics/pharmacodynamics of micafungin against *Candida* species in obese, critically ill, and morbidly obese critically ill patients

**DOI:** 10.1186/s13054-018-2019-8

**Published:** 2018-04-15

**Authors:** Emilio Maseda, Santiago Grau, Sonia Luque, Maria-Pilar Castillo-Mafla, Alejandro Suárez-de-la-Rica, Ana Montero-Feijoo, Patricia Salgado, Maria-Jose Gimenez, Carlos A. García-Bernedo, Fernando Gilsanz, Jason A. Roberts

**Affiliations:** 1 Department of Anesthesia and Surgical Intensive Care, Hospital Universitario La Paz, Paseo de la Castellana 261, 28046 Madrid, Spain; 2 Universidad Autónoma de Madrid, Madrid, Spain; 3 Pharmacy Department, Hospital del Mar, Barcelona, Spain; 4 Institut Hospital del Mar d’Investigacions Mèdiques (IMIM), Barcelona, Spain; 5 Universitat Autónoma de Barcelona, Barcelona, Spain; 6 PRISM-AG, Madrid, Spain; 7 Anesthesiology Department, Hospital del Mar, Barcelona, Spain; 8 Department of Molecular and Clinical Pharmacology, University of Liverpool, Liverpool, UK; 9 Burns, Trauma and Critical Care Research Centre, The University of Queensland, Brisbane, Australia; 10 Department of Intensive Care Medicine, Royal Brisbane and Women’s Hospital, Brisbane, Australia; 11 Pharmacy Department, Royal Brisbane and Women’s Hospital, Brisbane, Australia

**Keywords:** Morbid obesity, PK/PD, Monte-Carlo simulation, Intensive care unit, *Candida* spp.

## Abstract

**Background:**

Dosing in obese critically ill patients is challenging due to pathophysiological changes derived from obesity and/or critical illness, and it remains fully unexplored. This study estimated the micafungin probability of reaching adequate 24-h area under the curve (AUC_0–24h_)/minimum inhibitory concentration (MIC) values against *Candida* spp. for an obese/nonobese, critically ill/noncritically ill, large population.

**Methods:**

Blood samples for pharmacokinetic analyses were collected from 10 critically ill nonobese patients, 10 noncritically ill obese patients, and 11 critically ill morbidly obese patients under empirical/directed micafungin treatment. Patients received once daily 100–150 mg micafungin at the discretion of the treating physician following the prescribing information and hospital guidelines. Total micafungin concentrations were determined by high-performance liquid chromatography (HPLC). Monte-Carlo simulations were performed and the probability of target attainment (PTA) was calculated using the AUC_0–24_/MIC cut-offs 285 (*C. parapsilosis*), 3000 (all *Candida* spp.), and 5000 (non*parapsilosis Candida* spp.). Intravenous once-daily 100-mg, 150-mg, and 200-mg doses were simulated at different body weights (45, 80, 115, 150, and 185 kg) and age (30, 50, 70 and 90 years old). PTAs ≥ 90% were considered optimal. Fractional target attainment (FTA) was calculated using published MIC distributions. A dosing regimen was considered successful if the FTA was ≥ 90%.

**Results:**

Overall, 100 mg of micafungin was once-daily administered for nonobese and obese patients with body mass index (BMI) ≤ 45 kg/m^2^ and 150 mg for morbidly obese patients with BMI > 45 kg/m^2^ (except two noncritically ill obese patients with BMI ~ 35 kg/m^2^ receiving 150 mg, and one critically ill patient with BMI > 45 kg/m^2^ receiving 100 mg). Micafungin concentrations in plasma were best described using a two-compartment model. Weight and age (but not severity score) were significant covariates and improved the model. FTAs > 90% were obtained against *C. albicans* with the 200 mg/24 h dose for all body weights (up to 185 kg), and with the 150 mg/24 h for body weights < 115 kg, and against *C. glabrata* with the 200 mg/24 h dose for body weights < 115 kg.

**Conclusion:**

The lack of adequacy for the 100 mg/24 h dose suggested the need to increase the dose to 150 mg/24 h for *C. albicans* infections. Further pharmacokinetic/pharmacodynamic studies should address optimization of micafungin dosing for nonalbicans *Candida* infections.

## Background

Obesity, which is increasing at an alarming rate in developed countries, is a significant risk factor for nosocomial infections, especially following surgery due to the immune dysfunction associated with obesity [1]. In addition, pathophysiological changes in obese patients (e.g., reduced regional blood flow, altered cardiac output, increased fat and lean mass, etc.) might modify the pharmacokinetic/pharmacodynamic (PK/PD) profile of antimicrobials [2, 3]. On the other hand, critically ill patients also present pathophysiological changes (hepatic and/or renal dysfunction, hypoalbuminemia or increased capillary permeability, use of organ support modalities) that can alter antimicrobial clearance and volume of distribution [4]. Thus, dosing in obese critically ill patients is a challenging scenario for intensivists that has not been fully explored [5].

Micafungin is an echinocandin, a lipopeptide that exhibits concentration-dependent fungicidal activity against most species of *Candida* [6], and is licensed as a first-line treatment for invasive candidiasis [7]. The recently published study EUROBACT was conducted in 162 intensive care units (ICUs) in 24 countries. It showed that, among patients with candidemia, *Candida albicans* was the most frequent fungi isolated (57.1%), followed by *Candida glabrata* (15.3%), *Candida parapsilosis* (10.2%), and *Candida tropicalis* (6.1%) [8].

Altered serum concentrations of micafungin associated with morbid obesity in critically ill patients might impact the achievement of therapeutic drug exposures as defined by the area under the serum concentration curve over a 24-h period (AUC_0–24h_)/minimum inhibitory concentration (MIC), the pharmacodynamic index linked to clinical efficacy for micafungin [9, 10]. A previous study conducted by our group showed cumulative fraction responses > 90% for micafungin at the standard dose (100 mg) against *C. albicans* and *C. glabrata* in a special population of critically ill patients on continuous venovenous hemofiltration [11].

The aim of this study was to estimate the micafungin probability of achieving adequate AUC_0–24h_/MIC values against *Candida* spp. for a large population using Monte-Carlo simulations [10] and data from obese, critically ill, and morbidly obese critically ill patients treated with micafungin.

## Methods

A pharmacokinetic study was carried out in patients under micafungin empirical or directed treatment for invasive candidiasis. The population consisted of 11 morbidly obese critically ill adult patients (from the Hospital Universitario La Paz, Madrid, Spain), 10 nonobese critically ill patients, and 10 obese noncritically ill patients (from the Hospital del Mar, Barcelona, Spain). Patients admitted to ICUs were those considered to be critically ill. The study protocol was approved by the Ethics Committee of the Hospital La Paz (Madrid, Spain) and the Hospital del Mar (Barcelona, Spain). Written informed consent was obtained from patients (or relatives if the patient was unable to provide due to their critical situation) before blood sampling.

Demographic and clinical data prior to initiation of antifungal treatment were collected. Severity (Simplified Acute Physiology Score (SAPS) II) [12], Sequential Organ Failure Assessment (SOFA) score [13], and risk for invasive candidiasis (*Candida* score) [14] (except for patients with microbiologically documented infections) were calculated. Patients received dosage regimens of once-daily 100 mg or 150 mg micafungin (Astellas Pharma S.A., Spain) diluted in 100 ml isotonic saline solution that was intravenously infused over 60 min at the discretion of the treating physician (following the prescribing information and hospital treatment guidelines). On day 3, blood samples were collected at baseline (predose) and after 1, 3, 5, 8, 18, and 24 h. Additional blood samples at day 0 and day 7 were collected when feasible.

### Sample handling and storage

Blood samples were immediately placed on ice and centrifuged at 3000 rpm for 10 min. Following on, they were stored at −80 °C. The samples were transported by a commercial courier company to the Burns Trauma and Critical Care Research Centre, The University of Queensland, Australia, for further analysis.

### Drug assay

Total micafungin concentrations in plasma were measured by a validated ultra-high-performance liquid chromatography (UHPLC)-tandem mass spectrometry (MS/MS) method, from 0.2 to 30 μg/ml, on a Shimadzu Nexera 2 UHPLC system coupled to a Shimadzu 8030+ triple quadruple mass spectrometer (Shimadzu, Kyoto, Japan) [15]. Clinical samples were assayed alongside plasma calibrators and quality controls and met batch acceptance criteria [16].

### Generation of large population data

#### Population pharmacokinetic modeling

To describe total micafungin concentrations, one- and two-compartment models were developed with the nonparametric adaptive grid algorithm within the freely available Pmetrics software package for R (Los Angeles, CA, USA) [17, 18]. Elimination from the central compartment, and intercompartmental distribution into the peripheral compartment (two-compartment model), were modeled as first-order processes. The discrimination between different models resulted from the comparison of the −2 log-likelihood (−2LL). A *p* value of < 0.05 was considered statistically significant.

#### Population pharmacokinetics covariate screening

Age, gender, body weight, body mass index (BMI), Acute Physiology and Chronic Health Evaluation (APACHE) II, serum creatinine concentration, measured creatinine clearance, Cockroft-Gault estimated creatinine clearance, and serum albumin concentration were evaluated as covariates. Covariate selection was performed using a stepwise linear regression from R on all covariates and Bayesian posterior parameters. Potential covariates were separately entered into the model and statistically tested by use of the –2LL values. If inclusion of the covariate resulted in a statistically significant improvement in the LL values (*p* < 0.05) and in an improvement of the goodness-of-fit plots, then the covariate was retained in the final model.

#### Model diagnostics

Goodness-of-fit was assessed by linear regression, with an observed-predicted plot, coefficients of determination, and LL values. Predictive performance evaluation was based on mean error of prediction (bias) and mean bias-adjusted squared error of prediction (imprecision) of the population and individual prediction models. The internal validity of the population pharmacokinetic model was assessed by the bootstrap resampling method (*n* = 1000) and normalized prediction distribution errors (NPDE) [19]. Using a visual predictive check method, parameters obtained from the bootstrap method were plotted with the observed concentrations. NPDE plots were checked for normal distribution characteristics and trends in data errors [19].

#### Probability of target attainment (PTA)

Monte-Carlo simulations (*n* = 1000) were employed using Pmetrics software to determine the PTA of achieving the PK/PD target of AUC_0–24_/MIC (285 for *C. parapsilosis*, 3000 for all *Candida* spp., and 5000 for non*parapsilosis Candida* spp.) [11] for varying MICs (0.008 to 1 μg/ml). Intravenous once-daily doses of 100 mg, 150 mg, and 200 mg were simulated at different body weight (45, 80, 115, 150, and 185 kg) and age (30, 50, 70, and 90 years old). PTAs ≥ 90% were considered optimal.

#### Fractional target attainment (FTA) calculation

Published MIC distribution data of *C. parapsilosis*, non*parapsilosis Candida* spp., and all *Candida* spp. from the SENTRY study [20] were used to determine the FTA, which identifies the potential success of the treatment by comparing the pharmacodynamic exposure (i.e, PTA) against an MIC distribution. Specifically, PTA values determined at each MIC were multiplied by the fraction of isolates found at that MIC, and the sum of the products equaled the FTA. A value of FTA ≥ 90% against a population of organisms was considered optimal.

### Statistical analysis

Correlations were assessed by means of scatter graphs and the Pearson correlation coefficient (*r*).

## Results

Table 1 shows the demographic data, baseline analytical parameters, and clinical scores for the patients distributed by obese/nonobese and critically/noncritically ill categorization. Overall, the standard 100-mg dose of micafungin was once-daily administered for nonobese and obese patients with BMI ≤ 45 kg/m^2^. The 150-mg dose was administered for morbidly obese patients with BMI > 45 kg/m^2^, with the exception of two noncritically ill obese patients with BMI of around 35 kg/m^2^ who received the 150-mg dose, and one critically ill patient with BMI > 45 kg/m^2^ who received the 100-mg dose.

**Table 1:** Demographic and clinical data

Variable	Total	Critically morbidly obese		Noncritically obese		Critically nonobese
Dose (mg)		100	150	100	150	100
*n*	31	7	4	7	3	10
Age (years), median (range)	58 (27–85)	45 (27–73)	53.5 (44–63)	58 (48–85)	58 (43–73)	72 (43–85)
% Females	71.0	100	50.0	85.7	100	40
BMI^a^ (kg/m^2^), median (range)	34.7 (19.6–60.0)	44.2 (40.3–51.7)	52.8 (47.4–60.0)	27.7 (25.2–34.7)	35.5 (34.7–52.4)	23.1 (19.6–38.5)
Weight (kg), median (range)	95 (44–193)	113 (95–121)	157.5 (142–170)	84.1 (62–105)	105 (105–193)	65 (44.0–92.5)
Creatinine (mg/dl), median (range)	1.0 (0.4–3.9)	0.7 (0.6–1.5)	1.3 (0.6–1.7)	1.1 (0.7–1.4)	0.8 (0.7–1.5)	1.0 (0.4–3.0)
CrCl^a^ (ml/min/1.73m^2^)	93.4 ± 51.4	133.1 ± 44.9	112.5 ± 53.2	62.7 ± 38.4	105.4 ± 63.9	75.9 ± 46.1
Albumin (g/dl), median (range)	3 (1.2–4.0)	2.7 (1.9–3.5)	3.1 (2.4–4)	3.5 (2.6–3.9)	3.6 (3.4–3.7)	2.5 (1.2–3.3)
*Candida* score, median (range)	3 (2–4)^b^	3 (2–4)	3.5 (3–4)	DT^c^	DT^d^	3 (2–4)
SOFA^a^ score, median (range)	6 (0–12)	6 (2–8)	7 (5–10)	5 (2–10)	6 (5–7)	4.5 (0–12)
SAPS^a^ II, median (range)	34 (9–57)	40 (8–57)	34.5 (25–45)	47 (9–53)	41 (18–44)	26 (11–42)

Data are expressed as mean ± standard deviation, except where stated

^a^BMI, Body mass index; CrCl, creatinine clearance; SAPS, Simplified Acute Physiology Score; SOFA, Sequential Organ Failure Assessment

^b^ For 21 patients

^c^ Directed treatment (DT): three candidemia, three osteoarticular infections, and one urinary tract infection

^d^ DT: one peritonitis, one urinary tract infection, and one osteoarticular infection

**Fig. 1:**
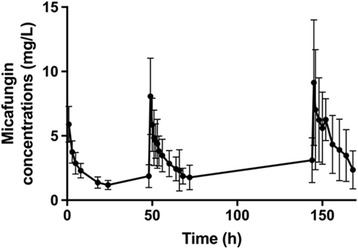
Micafungin concentrations. Mean observed micafungin concentration-time profiles (error bars represent standard deviation)

Figure 1 shows the mean observed concentration-time profile of micafungin concentrations for the study population.

### Pharmacokinetic model

A two-compartment linear model (including zero order input of drug into the central compartment) best described the time course of 242 total plasma concentrations of micafungin. The goodness-of-fit of the model was improved (*p* < 0.05) by the inclusion of the covariate body weight (normalized to 70 kg) and age (normalized to 60 years old to an exponential value of 0.75) for micafungin clearance. Use of this exponent on age improved the model better than either covariate added as a linear function alone and reflected the likely nonlinear effect of the increasing body weight and age on micafungin clearance. Addition of body weight or age alone did not statistically improve the model when compared with the structural model (− 2LL value, 595.2 vs 596.4 for weight inclusion, *p* = 0.0586; 587.5 vs 596.4 for age inclusion, *p* = 0.597). When both the covariates body weight and age were included, the log likelihood value decreased significantly (−2LL, 415.6; *p* = 0.0238) and the goodness-of-fit of the model also showed an improvement. The final covariate model was as follows:

$$ \mathrm{Micafungin}\ \mathrm{CL}={\mathrm{TVCL}}_{\ast }{{\left(\mathrm{Wt}/70\right)}^{0.75}}_{\ast }{\left(\mathrm{Age}/60\right)}^{0.75} $$

Where TVCL is the typical value of micafungin clearance, Wt is the total body weight (kg) and Age is the patient’s age (years).

**Fig. 2:**
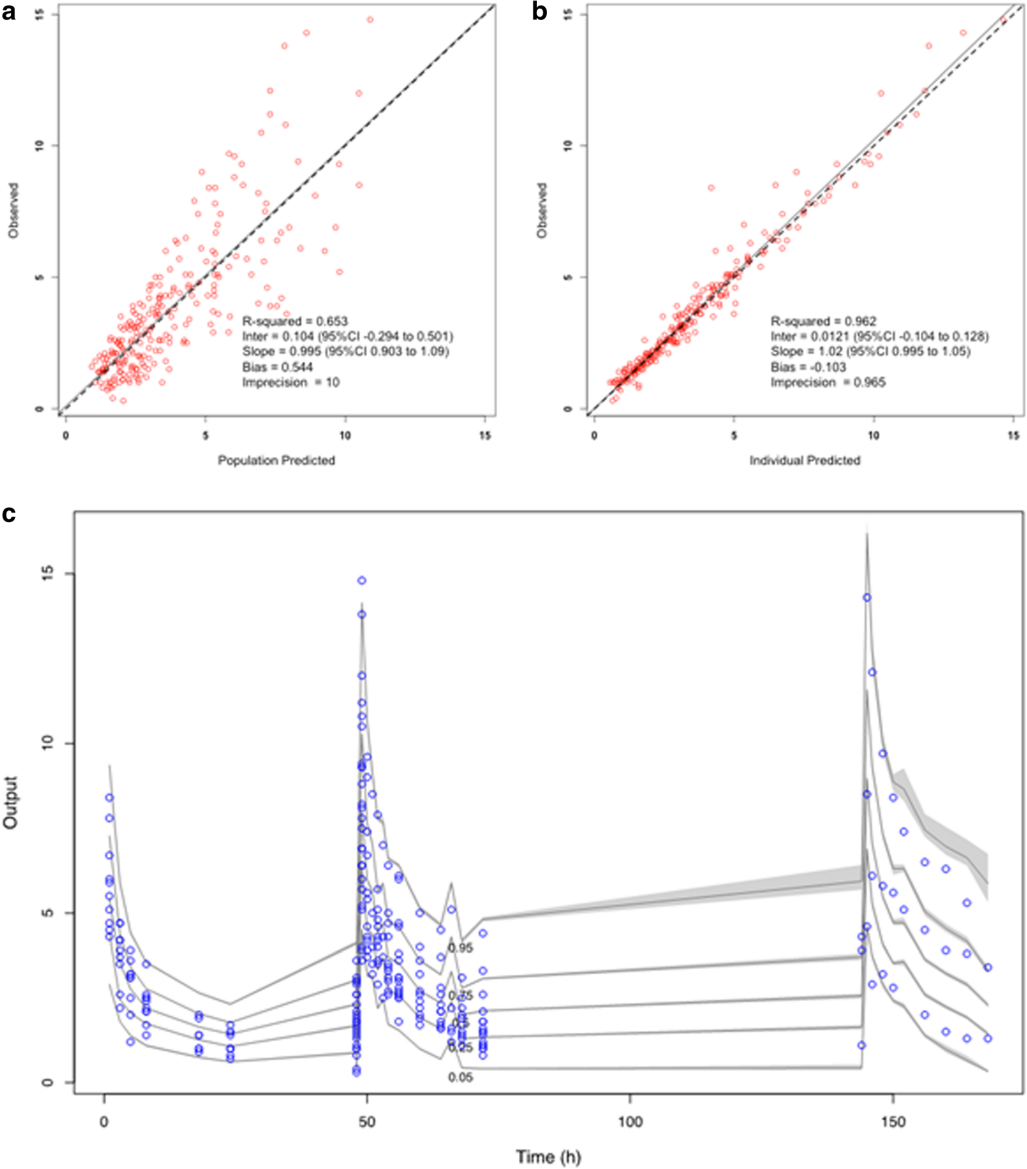
Diagnostic plots for the final population pharmacokinetic covariate model. **a** Observed micafungin concentrations versus population predicted concentrations. **b** Observed micafungin concentrations versus individual predicted concentrations. **c** Visual predictive check (circles represent observed data). Concentrations are expressed as μg/ml

The mean ± standard deviation (SD) population pharmacokinetic parameter estimates for the final covariate model are shown in Table 2. The diagnostic plots confirmed the appropriateness of the model as shown in Fig. 2. The final covariate model was then used for Monte-Carlo dosing simulations.

**Table 2:** Estimated micafungin parameters

Parameters	Mean ± SD	Coefficient of variation (%)	Variance	Median
Clearance (l/h)	0.80 ± 0.49	61.78	0.24	0.73
Central volume (l)	16.34 ± 5.87	35.95	34.49	16.34
*k*cp (h^−1^)	0.38 ± 0.37	97.76	0.14	0.26
*k*pc (h^−1^)	0.32 ± 0.31	96.51	0.09	0.14

Parameter estimates for micafungin from the final two-compartment covariate population pharmacokinetic model

*kcp*, rate constant for drug distribution from the central to peripheral compartment; *k*pc, rate constant for drug distribution from the peripheral to central compartment

### Dosing simulations

PTAs for AUC_0–24_/MIC of 285, 3000, or 5000 for different micafungin doses (100 mg, 150 mg, 200 mg) and body weights (from 45 kg to 185 kg) for patients with a medium age of 70 years old (no significant changes were observed in simulations with different patient ages) are described in Table 3. The Monte-Carlo simulations showed that increases in the micafungin dose resulted in increased PTAs. For non*parapsilosis Candida* (AUC/MIC > 5000) this target attainment was only obtained with > 90% probability with doses of 150 mg and 200 mg against isolates with MICs up to 0.008 μg/ml, regardless of the patient’s weight.

**Table 3:** Probability of target attainment (PTA) for micafungin

		AUC_0–24_/MIC of 285 for body weight (kg) equal to:					AUC_0–24_/MIC of 3000 for body weight (kg) equal to:					AUC_0–24_/MIC of 5000 for body weight (kg) equal to:				
Dose (mg/24 h)	MIC (μg/ml)	45	80	115	150	185	45	80	115	150	185	45	80	115	150	185
100	0.008	100	100	100	100	100	99.0	98.9	98.8	98.5	97.8	83.7	78.4	72.3	64.0	55.1
	0.016	100	100	100	100	100	72.0	58.8	52.2	40.9	31.1	9.2	3.7	1.4	0.5	0.2
	0.032	100	100	100	100	100	23.0	0.7	0.1	0.1	0.0	0.0	0.0	0.0	0.0	0.0
	0.064	99.9	99.7	99.7	99.7	99.6	0.0	0.0	0.0	0.0	0.0	0.0	0.0	0.0	0.0	0.0
	0.125	93.8	85.6	79.0	75.8	71.2	0.0	0.0	0.0	0.0	0.0	0.0	0.0	0.0	0.0	0.0
	0.25	23.4	9.8	4.8	1.9	0.8	0.0	0.0	0.0	0.0	0.0	0.0	0.0	0.0	0.0	0.0
	0.5	0.0	0.0	0.0	0.0	0.0	0.0	0.0	0.0	0.0	0.0	0.0	0.0	0.0	0.0	0.0
	1	0.0	0.0	0.0	0.0	0.0	0.0	0.0	0.0	0.0	0.0	0.0	0.0	0.0	0.0	0.0
150	0.008	100	100	100	100	100	100	100	100	99.9	99.8	98.8	98.4	98.1	97.2	91.3
	0.016	100	100	100	100	100	96.7	95.5	89.6	80.8	78.6	57.3	49.2	35.4	25.3	17.6
	0.032	100	100	100	100	100	38.5	22.2	12.3	6.9	3.0	0.9	0.1	0.1	0.0	0.0
	0.064	100	100	100	100	100	0.1	0.0	0.0	0.0	0.0	0.0	0.0	0.0	0.0	0.0
	0.125	99.1	98.9	98.9	98.6	98.0	0.0	0.0	0.0	0.0	0.0	0.0	0.0	0.0	0.0	0.0
	0.25	72.8	59.8	52.8	42.7	32.5	0.0	0.0	0.0	0.0	0.0	0.0	0.0	0.0	0.0	0.0
	0.5	2.5	0.8	0.1	0.1	0.0	0.0	0.0	0.0	0.0	0.0	0.0	0.0	0.0	0.0	0.0
	1	0.0	0.0	0.0	0.0	0.0	0.0	0.0	0.0	0.0	0.0	0.0	0.0	0.0	0.0	0.0
200	0.008	100	100	100	100	100	100	100	100	100	100	99.7	99.7	99.6	99.4	99.1
	0.016	100	100	100	100	100	99.0	98.9	98.8	98.5	97.8	83.7	78.4	72.3	64.0	55.1
	0.032	100	100	100	100	100	72.0	58.8	52.2	40.9	31.1	9.2	3.7	1.4	0.5	0.2
	0.064	100	100	100	100	100	2.3	0.7	0.1	0.1	0.0	0.0	0.0	0.0	0.0	0.0
	0.125	99.9	99.8	99.7	99.7	99.7	0.0	0.0	0.0	0.0	0.0	0.0	0.0	0.0	0.0	0.0
	0.25	93.8	85.6	79.0	75.8	71.2	0.0	0.0	0.0	0.0	0.0	0.0	0.0	0.0	0.0	0.0
	0.5	23.4	9.8	4.8	1.9	0.8	0.0	0.0	0.0	0.0	0.0	0.0	0.0	0.0	0.0	0.0
	1	0.0	0.0	0.0	0.0	0.0	0.0	0.0	0.0	0.0	0.0	0.0	0.0	0.0	0.0	0.0

Micafungin PTA for different target values of area under the serum concentration curve over a 24-h period divided by the minimum inhibitory concentration (AUC_0–24_/MIC), body weights, and once-daily doses

FTAs for the simulated PTAs against MIC distributions for *C. albicans*, *C. glabrata*, *C. parapsilosis*, and *C. tropicalis* are shown in Table 4. FTAs > 90% were obtained against *C. albicans* with the 200 mg/24 h dose for all body weights, and with the 150 mg/24 h dose for body weights of 45 kg, 80 kg, and 115 kg, and against *C. glabrata* for body weights of 45 kg, 80 kg, and 115 kg with the 200 mg/24 h dose. No FTAs > 90% were obtained with the 100 mg/24 h dose regardless of the species or the patient’s weight.

**Table 4:** Fractional target attainment (FTA) for micafungin

	100 mg/24 h					150 mg/24 h					200 mg/24 h				
Body weight (kg)	45	80	115	150	185	45	80	115	150	185	45	80	115	150	185
*C. albicans*	74.6	62.6	56.6	46.4	37.4	97.0	95.9	90.6	82.6	80.6	99.1	99.0	98.9	98.6	98.0
*C. glabrata*	62.0	52.3	47.5	39.3	32.2	86.4	82.6	76.5	69.3	67.0	94.2	91.7	90.4	88.1	85.8
*C. tropicalis*	23.9	19.3	17.1	15.9	11.0	49.6	40.8	34.1	28.8	26.1	67.9	60.8	57.3	51.4	46.1
*C. parapsilosis*	1.6	1.2	1.0	0.9	0.9	3.6	2.7	2.4	2.2	1.9	6.6	4.6	3.7	3.3	3.0

Micafungin FTA calculated using MIC distributions [20] for the different *Candida* species

## Discussion

The present Monte-Carlo simulation using data from obese, critically ill, and morbidly obese critically ill patients treated with micafungin estimated the micafungin probability of achieving adequate AUC_0–24h_/MIC values against *Candida* spp. for a large population. Our results showed the lack of adequate micafungin exposure (in terms of FTAs) with the 100 mg/24 h dose regardless of the *Candida* species or the patient’s weight. Against *C. albicans*, micafungin exposure was adequate with the 150 mg/24 h dose for patients weighing up to 115 kg and with the 200 mg/24 h dose for those surpassing such a weight.

As in previous studies, plasma concentrations of micafungin were best described using a two-compartment model and, as mentioned, weight was a significant covariate [21]. Unlike the introduction of the severity score as a covariate, introducing the patient’s age improved the model. The influence of severity scores on micafungin exposure in severely ill patients is controversial among studies in the literature; while one study considered SOFA as a relevant covariate [22], another study did not find a correlation of APACHE II or SOFA with exposure, suggesting the possibility of being ruled out as a cause of low drug exposure [23]. The reason for this could be the high interindividual variability found in studies investigating micafungin exposure in critically ill patients [23–25] in contrast to data from healthy volunteers or patients under continuous venovenous hemofiltration [11, 24], which represent more uniform populations.

On the contrary, weight has been described as markedly influencing micafungin clearance [21] both in patients weighing > 66.3 kg [26] and in healthy volunteers in a study including subjects with BMI < 25, 25–40, and > 40 kg/m^2^ [27, 28]. In the present simulation, the inclusion of weight as a covariate improved the model. According to the results of our model, for MIC values > 0.008 μg/ml, the 100 mg/24 h dose failed to achieve the optimal ratio threshold of AUC_0–24h_/MIC of 3000. This cut-off was associated with therapeutic outcome in animal models for disseminated candidiasis by *C. albicans* [29], and it was extrapolated to humans [9] using data from clinical trials of invasive candidiasis/candidemia [30]. The increase in micafungin exposure provided by the doses of 150 or 200 mg/24 h markedly improved the coverage encompassing MICs of 0.016 μg/ml for body weights up to 80 kg (with the 150 mg/24 h dose) or for all body weights (200 mg/24 h dose). When considering recent MIC distributions for *C. albicans*, the most frequent isolated species, our results indicate that, to obtain adequate coverage (FTA ≥ 90%), the micafungin dose should be increased to 150 mg/24 h for nonobese patients (≤ 115 kg) and to 200 mg/24-h for those with body weight > 115 kg. This finding is consistent with previous reports showing the need for an increase in doses of antifungals in obese patients [5, 15, 31, 32] due to inadequate exposure with standards doses, as described with micafungin [26, 33]. In this sense, there is a report suggesting inadequate exposure with micafungin 100 mg/24 h in an obese critically ill patient weighing 230 kg [34]. Exposure may be crucial in morbidly obese critically ill patients when compared with the ICU population or obesity alone [32]. A timely and sufficiently high exposure to the appropriate antifungal agent is essential for the eradication of the pathogen. This acquires importance since, worldwide, mean weight, both in men and women, has been increasing over the last decades. In the USA, during 2013 and 2014, the overall age-adjusted prevalence of obesity was 37.7% [35].

Increasing the dose to 200 mg/24 h would overcome the problem caused by being overweight for *C. albicans*; however, such an increase would not solve the problem for other *Candida* species, requiring higher exposures. Since a previous study indicated that the maximum tolerated dose of micafungin in patients undergoing hematopoietic stem cell transplantation was at least up to 8 mg/kg/24 h [36], strategies including individualized dosing have been advocated as a great opportunity to further improve the efficacy of micafungin [27], an antifungal with reported 70–80% efficacy in the treatment of candidemia with the current dosing of 100 mg/24 h [27, 30, 37, 38].

The results of this study are of high importance due to the very limited information available on the pharmacokinetics and efficacy of echinocandins in obese critically ill patients, especially in those with morbid obesity. Most studies have been performed with caspofungin. In agreement with our results, pharmacokinetic studies with caspofungin showed lower exposure in overweight and obese patients, whether critically ill [39] or not [40], and also showed the benefits of increasing the dose in morbidly obese patients [41]. Similarly, the limited data in the literature regarding the influence of obesity on the pharmacokinetics of anidulafungin confirm the lower anidulafungin exposure in patients with morbid obesity compared with nonobese patients [32] and the need for increasing the dose in a critically ill morbid obese patient [42].

The present study is the first population assessment of micafungin in critically ill nonobese, noncritically ill obese, and critically ill morbidly obese patients. Several limitations and challenges must be kept in mind in this respect. Despite the relatively large sample size in this study, the distribution of patients resulted in a low number of individuals in some of the groups. In addition, the present study is a pharmacodynamic modeling not designed to examine the effect of micafungin exposure on patient outcome; clinical trials should address this issue from a clinical perspective.

## Conclusion

The results of this study indicate that micafungin exposure was adequate with the 150 mg/24 h dose for patients weighing up to 115 kg and with the 200 mg/24 h dose for those surpassing such weight to cover *C. albicans*. The 200 mg/24 h dose covered *C. glabrata* for patients weighing up to 115 kg. Since other species of *Candida* were not successfully covered, further PK/PD studies should address this point to optimize dosing for nonalbicans *Candida* infections.

## Abbreviations

APACHE: Acute Physiology and Chronic Health Evaluation
AUC: Area under the curve
BMI: Body mass index
FTA: Fractional target attainment
ICU: Intensive care unit
LL: Log-likelihood
MIC: Minimum inhibitory concentration
MS/MS: Tandem mass spectrometry
NPDE: Normalized prediction distribution errors
PK/PD: Pharmacokinetic/pharmacodynamic
PTA: Probability of target attainment
SAPS: Simplified Acute Physiology Score
SD: Standard deviation
SOFA: Sequential Organ Failure Assessment
UHPLC: Ultra-high-performance liquid chromatography
